# The role of superficial and deep layers in the generation of high frequency oscillations and interictal epileptiform discharges in the human cortex

**DOI:** 10.1038/s41598-022-22497-2

**Published:** 2023-06-14

**Authors:** Daniel Fabo, Virag Bokodi, Johanna-Petra Szabó, Emilia Tóth, Pariya Salami, Corey J. Keller, Boglárka Hajnal, Thomas Thesen, Orrin Devinsky, Werner Doyle, Ashesh Mehta, Joseph Madsen, Emad Eskandar, Lorand Erőss, István Ulbert, Eric Halgren, Sydney S. Cash

**Affiliations:** 1 Epilepsy Unit, Department of Neurology, National Institute of Mental Health, Neurology and Neurosurgery, Amerikai Út 57. 1145, Budapest, Hungary; 2 Roska Tamás Doctoral School of Sciences and Technologies, Budapest, Hungary; 3 János Szentágothai Doctoral School of Neurosciences, Budapest, Hungary; 4 Department of Neurology, University of Texas, McGovern Medical School, Houston, TX USA; 5 Epilepsy Division, Department of Neurology, Department of Neurosurgery, Massachusetts General Hospital, Harvard Medical School, Boston, MA USA; 6 Department of Psychiatry and Behavioral Sciences, Stanford University Medical Center, Stanford, CA USA; 7 VA Palo Alto Health Care System, Palo Alto, CA USA; 8 Comprehensive Epilepsy Center, New York University School of Medicine, New York, NY USA; 9 Department of Biomedical Sciences, College of Medicine, University of Houston, Houston, TX USA; 10 Department of Neurosurgery, Zucker School of Medicine at Hofstra/Northwell and Feinstein Institute for Medical Research, Manhasset, NY USA; 11 The Children’s Hospital, Boston, MA USA; 12 Massachusetts General Hospital Neurosurgery Research, Boston, MA USA; 13 Department of Functional Neurosurgery, National Institute of Mental Health, Neurology and Neurosurgery, Budapest, Hungary; 14 Institute of Psychology, Eötvös Loránd Research Network, Budapest, Hungary; 15 Department of Radiology, Neurosciences and Psychiatry, University of California, San Diego, San Diego, CA USA

**Keywords:** Neuroscience, Medical research, Neurology

## Abstract

Describing intracortical laminar organization of interictal epileptiform discharges (IED) and high frequency oscillations (HFOs), also known as ripples. Defining the frequency limits of slow and fast ripples. We recorded potential gradients with laminar multielectrode arrays (LME) for current source density (CSD) and multi-unit activity (MUA) analysis of interictal epileptiform discharges IEDs and HFOs in the neocortex and mesial temporal lobe of focal epilepsy patients. IEDs were observed in 20/29, while ripples only in 9/29 patients. Ripples were all detected within the seizure onset zone (SOZ). Compared to hippocampal HFOs, neocortical ripples proved to be longer, lower in frequency and amplitude, and presented non-uniform cycles. A subset of ripples (≈ 50%) co-occurred with IEDs, while IEDs were shown to contain variable high-frequency activity, even below HFO detection threshold. The limit between slow and fast ripples was defined at 150 Hz, while IEDs’ high frequency components form clusters separated at 185 Hz. CSD analysis of IEDs and ripples revealed an alternating sink-source pair in the supragranular cortical layers, although fast ripple CSD appeared lower and engaged a wider cortical domain than slow ripples MUA analysis suggested a possible role of infragranularly located neural populations in ripple and IED generation. Laminar distribution of peak frequencies derived from HFOs and IEDs, respectively, showed that supragranular layers were dominated by slower (< 150 Hz) components. Our findings suggest that cortical slow ripples are generated primarily in upper layers while fast ripples and associated MUA in deeper layers. The dissociation of macro- and microdomains suggests that microelectrode recordings may be more selective for SOZ-linked ripples. We found a complex interplay between neural activity in the neocortical laminae during ripple and IED formation. We observed a potential leading role of cortical neurons in deeper layers, suggesting a refined utilization of LMEs in SOZ localization.

## Introduction

The success of surgical treatment in patients with drug resistant epilepsy (DRE) depends to a great degree on the accuracy with which the epileptogenic zone (EZ) can be localized during presurgical evaluation. Currently, the resection is guided by the identification of the seizure onset zone (SOZ), the region where clinical seizures initiate. However, the SOZ might not fully overlap with the EZ, the area “indispensable for the generation of epileptic seizures”^1^, which might lead to the failure of surgical intervention. Although in recent years, several novel biomarkers, derived from both invasive and noninvasive electrophysiological recordings, have been proposed to improve the delineation of the EZ and to predict surgical outcome, only a few of them proved to be useful in clinical practice^2,3^.

High frequency oscillations (HFOs) have emerged as promising biomarkers of the EZ^4,5^, however due to the heterogeneity of this phenomenon and the high variability among patients, it is still unclear how to definitively differentiate pathological HFOs from normally occurring oscillations^6,7^, hence recent findings have questioned the clinical usefulness of HFOs^8,9^. This controversy points to a need for better understanding of the underlying mechanisms of HFOs.

HFOs are brief bursts of high frequency (> 80 Hz) oscillations recorded with invasive and noninvasive EEG, which can be distinguished visually on the raw local field potential (LFP) signal^6,7,10^. It has to be noted, that high-frequency contents obtained by spectral decomposition do not necessarily reflect HFOs, as sharp transients, such as sharp waves or epileptic spikes, and certain artifacts increase power of high frequency components, without producing true signal oscillations^10^. Based on the frequency of oscillations HFOs can be divided into “slow ripples” (80–200 Hz) and “fast ripples” (> 150–250 Hz)^11,12^. Confusion arises from the partly overlapping terms (HFO, ripple, fast ripple). Thus, we will use the HFOs in general, and ‘slow ripple’ or ‘fast ripple’ selectively for visually detected and spectrum filtered oscillations.

HFOs were first described in non-human primates in the mesial temporal lobe^13^ associated with information transfer between the hippocampus and extra-hippocampal structures^14^. Subsequently, HFOs were found in animal models of epilepsy^11,15,16^, and in human epileptic brain regions^17–20^.

The precise role of HFOs in ictogenesis or epileptogenesis remains unclear despite extensive research^21^.^.^Epileptogenic processes may be associated with fast ripples^4,17,18^, as they correlate with the SOZ^4,22,23^, histopathological alterations^24^, surgical outcome^25,26^ and seizure propensity^27,28^. Interictal HFOs occur mostly in mesial temporal lobe^16^, but also occur in neocortical regions^19,29^ predominantly with focal cortical dysplasias (FCD)^4,30^; tumors, cortical tubers, and nodular heterotopia^31,32^. Interictal HFOs were considered more focal and specific than interictal epileptiform discharges (IED)^31,33^, but recent studies suggest that co-occurring HFOs and IEDs provide even greater specificity and sensitivity for epileptogenic regions^34,35^. The pathologic nature of HFOs cannot be determined solely by frequency. Based on a tetanus toxin induced neocortical epilepsy model, pathological neocortical HFOs may fall in the slower band (< 160 Hz)^36^. However, some neocortical areas are prone to generate fast ripples (> 200 Hz) in awake normal conditions like somatosensory stimulation^6^, which reflected millisecond synchronization of high-frequency neuronal firing^37,38^. Based on detailed analysis of HFO rates in non-epileptic cortices of epileptic patients there are maps available for slow and fast ripples under normal conditions^39^.

The neuronal mechanisms of HFO have been extensively studied leading to multiple hypotheses, including possible cellular alterations, like pathological gap junction connections^40^ and asynchronous firing of local neuronal groups^15,41^, including weakly synchronized pyramidal cells^42^. Despite these studies, little is known about the organization of neuronal sources for HFOs in patients with epilepsy. This problem is compounded by the heterogeneity in HFO appearance and function^39^, thus understanding the intracortical organization of patients’ HFOs would advance our ability to classify HFOs, potentially creating a more valid biomarker for epileptic regions and activity.

The laminar organization of various pathological and physiological brain activities, like IEDs^43^, cortical slow oscillations^44^, spindles^45,46^, or seizure initiation^47^ are shown to display specific patterns, resulting in the observation that cortical lamination has a huge impact on the generation of cortical oscillations and pathological activities. We also know that faster HFO activities are more pathological as a general rule. We hypothesized that these differences will be reflected in cortical layer involvement in these processes.

Our aim was to understand HFO generators in the epileptic brain. We employed laminar multi-microelectrodes in DRE patients^43^ to record field potential gradients and perform current source density (CSD), multiple unit analysis (MUA) and time frequency analysis for visually selected HFOs (80–500 Hz) and IEDs. We detected HFOs and IEDs separately and measured the associated high frequency spectral distribution throughout the cortical layers with time–frequency analysis methods. To our knowledge this study represents the first to examine the role of specific cortical laminae in HFO generation in humans.

## Methods

### Participants

Participants were recruited as part of an ongoing, multi-site collaborative project between 2003 and 2016 (Beth Israel Deaconess Medical Center, Brigham and Women’s Hospital, Children’s Hospital and Massachusetts General Hospital in Boston, and New York University Medical Center, New York and Epilepsy Center of National Institute of Mental Health, Neurology and Neurosurgery (former names: Natl. Inst. of Psychiatry and Neurology, Natl. Inst. of Clinical Neurosciences, Budapest, Hungary).

Forty-four patients with medically intractable epilepsy undergoing evaluation with subdural grid and/or intracerebral depth electrodes were implanted with the experimental micro-electrodes. Fifteen patients were excluded due to poor data quality, resulting in twenty-nine patients with good quality data (n = 29). Exclusion due to poor data quality was considered in two conditions. On one hand, when the amount of high frequency noise, overlapping with HFO frequency range, rendered HFO detection impossible (most of the excluded cases) and on the other hand, when the recording was abrupted on most of the channels due to technical issues, such as broken cables or faulty electrodes (Supplementary Fig. 1).

The choice of patients for intracranial studies, the location of the clinical electrodes, duration of the implantation and, ultimately, the excision of cortex, were determined entirely on clinical grounds by an independent clinical team without regard for experimental considerations. All patients gave written informed consent to the experimental procedure after a thorough explanation of the risks associated with the procedures monitored. All methods were approved by local Institutional Review Boards (Intézeti Kutatásetikai Bizottság, IKEB) and the National Ethical Board (TUKEB, Hungary). All method were carried out in accordance with the Declaration of Helsinki.

Determination of clinical etiology was made by the primary clinical team and, where possible, confirmed through pathological analysis of resected tissue.

### Implantation procedure and recording protocol

Laminar multielectrodes (LME) were implanted during the craniotomy or burr hole surgery of the patients when diagnostic intracranial electrodes (grids, strips or depth electrodes; together referred to as macroelectrodes) were implanted in order to study the epileptogenic zone (EZ). Macroelectrode positions were carefully planned by the neurologists and neurosurgeons based on presurgical electrophysiological and imaging data resulting in the best hypothesis of the EZ location. Macroelectrode planning and surgery was conducted following clinical protocols without impact from the present study. LME implantation was the last step of the implantation phase of the surgery, that was followed by the wound closing procedure. Although it could not be determined with full certainty, LMEs were intended to be placed within EZ. The final location of the LMEs was ultimately determined by the neurosurgeon, who combined the best available information on the EZ with safety considerations to cause the least possible harm to the patient, and the least disturbance in the ongoing surgical procedure (e.g. additional opening of the skull, or dislocation of the macroelectrodes were not accepted).

Strip and grid contacts were made of 3 mm platinum discs forming either 8 × 8 grid arrays or 1 × 8 strips, with 10 mm intercontact distances (Ad-Tech Medical Instrument Co., Racine, Wisconsin). Recording from the macroelectrode arrays was implemented by 128 channel clinical cable telemetry systems with sampling rate of 1024 Hz (Micromed S.p.A., Treviso, Italy; or Natus Medical Inc., Middleton, WI, USA).

Two types of laminar multielectrode arrays (LME) were employed depending on the clinical situation (details of electrode construction, recordings and analysis have been published previously^48^). Both arrays consisted of 24 Platinium/Iridium contacts with diameters of 40 μm and intercontact distances of 150 μm on centers arranged in a line. The contacts on each probe were exposed on the side of a polyimide tube with a total diameter of 350 μm and length of ~ 3.5 mm. The sliding of the first contact (with more than 100 μm) below the pial surface was impeded by a silicone sheet appended to the top of the microelectrode array shank.

The first electrode type, a thumbtack-like array, was a short laminar probe (Neuronelectrod Kft., Budaörs, Hungary) inserted into the gyral crown beneath a subdural grid electrode (n = 26). Care was taken to insert the multielectrode perpendicular to the cortical surface. The second type electrode array was inserted into the lumen of depth electrodes implanted in mesial temporal areas (Ad-Tech Medical Instrument Corporation, Racine, Wisconsin) and extended ~ 3.5 mm from the tip of that electrode (n = 3).

The relationship of the LME probe to the cortical lamina was determined by characteristic activity patterns: cortical surface is marked by a sudden drop of IED amplitude and an appearance of slow artifacts indicating the surrounding corticospinal fluid^44,46,48,49^.

Differential recordings were made from each pair of successive contacts to establish a potential gradient. Recording apparatus including amplifier and filter characteristics were reported^48–51^. Briefly, using a self-manufactured (IU) preamplifier and amplifier system, after wideband (DC-10000 Hz) preamplification (gain 10x, CMRR 90db, input impedance 10^12^ ohms), the signal was recorded (filtered at 0.2–1000 Hz, gain 1000×, digitized at 2000 Hz, 16 bit), and stored continuously co-recorded with the clinical electrodes using time-locked triggers.

Analyses were performed using Neuroscan Edit 4.3 software (Compumedics, El Paso, TX) and with custom designed MATLAB (MathWorks, Natick, MA), LabVIEW (National Instruments Corp., Austin, TX) and C/C++ codes, as well as publicly available software suites (EEGLAB)^52^.

### Selection of IEDs

IED detection was performed semi-automatically on daytime LFP traces based on typical morphological characteristics for sharp waves, spikes, and spike-wave discharges^53^. Artifact-free continuous segments were selected for each patient recorded > 2 h before or after a seizure. Baseline amplitude variance was determined on each channel separately, using the whole recording period First, IEDs were identified automatically, using a custom threshold-based algorithm. Activation was considered to be significant when the peak exceeded the 2.5 standard deviation (SD) limit compared to baseline, based on literature data and careful inspection of data prior analysis^54,55^. Then, each detected event was reviewed visually and those were selected for further analysis that showed biphasic or triphasic morphology with an initial fast phase of maximum 200 ms, followed by a prolonged, slower phase that lasted longer than 200 ms.

### Selection of HFOs

Detection of HFOs was based on the methods described by Staba and Crepon et al. and combined both automated and visual approaches^19,22^. After careful artifact rejection, putative HFO events were selected automatically by applying a 5SD threshold to the root-mean-square (RMS; 5 ms sliding window) transformed band-pass filtered (zero-phase shift 4th order Butterworth digital filter 80–500 Hz) data. To avoid the unnecessary detection of the same event recorded on multiple channels, co-occurring (within less than 100 ms) events were grouped on each multielectrode and the highest amplitude event in each group was selected for further inspection. These events were also visually reviewed by 2 epileptologists (DF, ET).

Wave parameters were calculated automatically according to Staba et al.^22^, using a time window of − 500 to + 500 ms relative to the peak amplitude of detected HFO events. Duration and number of cycles were determined by the length and number of detected peaks, respectively, of the RMS data exceeding the 3SD threshold^22^. Instantaneous frequency was calculated by dividing the number of cycles by the duration in seconds. Amplitude was expressed as z-score relative to the mean of total artifact-free band-pass filtered data.

The originally selected events were only accepted if they satisfied the following criteria: having at least 3 cycles above the 3SD duration window of RMS data and lasting for more than 6 ms. HFO density was given as the number of HFO events in every minute of the artifact-free period (rate/minute)^22^.

Since there is a confusion in the literature about the appropriate boundary separating slow and fast ripples, we have addressed this question, by testing multiple band-pass filters (zero-phase shift 4th order Butterworth digital filter) with consecutively increasing lower frequency thresholds (150, 200 and 250 Hz, respectively) to determine the optimal cutoff frequency for the differentiation of slow and fast oscillations. Filters with varying lower cutoff frequencies were applied to the same line of events (1 s time windows around peak amplitude of detected HFOs). Similarly, filtered events were subjected to the same selection criteria described above. Wave parameters and HFO density were also recalculated in these cases.

### CSD and MUA calculation

CSD is a powerful method to estimate the laminar distribution and time course of the sources and sinks of membrane currents from LFP recorded at different depths^56^. CSD was calculated from the second spatial derivative of the LFP signal (filtered at 80–150 Hz for slow ripples and 150–500 Hz for fast ripples and averaged across events), as described by Ulbert et al.^48^. Sinks and sources were considered significant if exceeded mean ± 5SD threshold. It has to be noted that CSD values were used only to extract Fig. 3a,c,e, all other analyses were performed on LFP data.

MUA, as a measure of population cellular activity, sheds light on the local cellular responses in relation with the LFP. MUA was obtained by further filtering and rectification (48 db/oct zero phase shift 4th order Butterworth band-pass filter at 500–1000 Hz) of the signal in a range found to detect action-potentials in previous studies^57^. A final continuous estimate of MUA is derived by passing the signal through a 50 Hz low pass digital filter (24 db/oct zero phase shift)^48^. MUA was averaged across slow and fast ripples separately. MUA significance was calculated from the mean MUA values channel by channel, applying the same methodology as in the case of CSD (mean MUA activity ± 5 SD).

### Time–frequency analysis

Two time–frequency methods were applied for high frequency spectral analysis.

**Figure 1:**
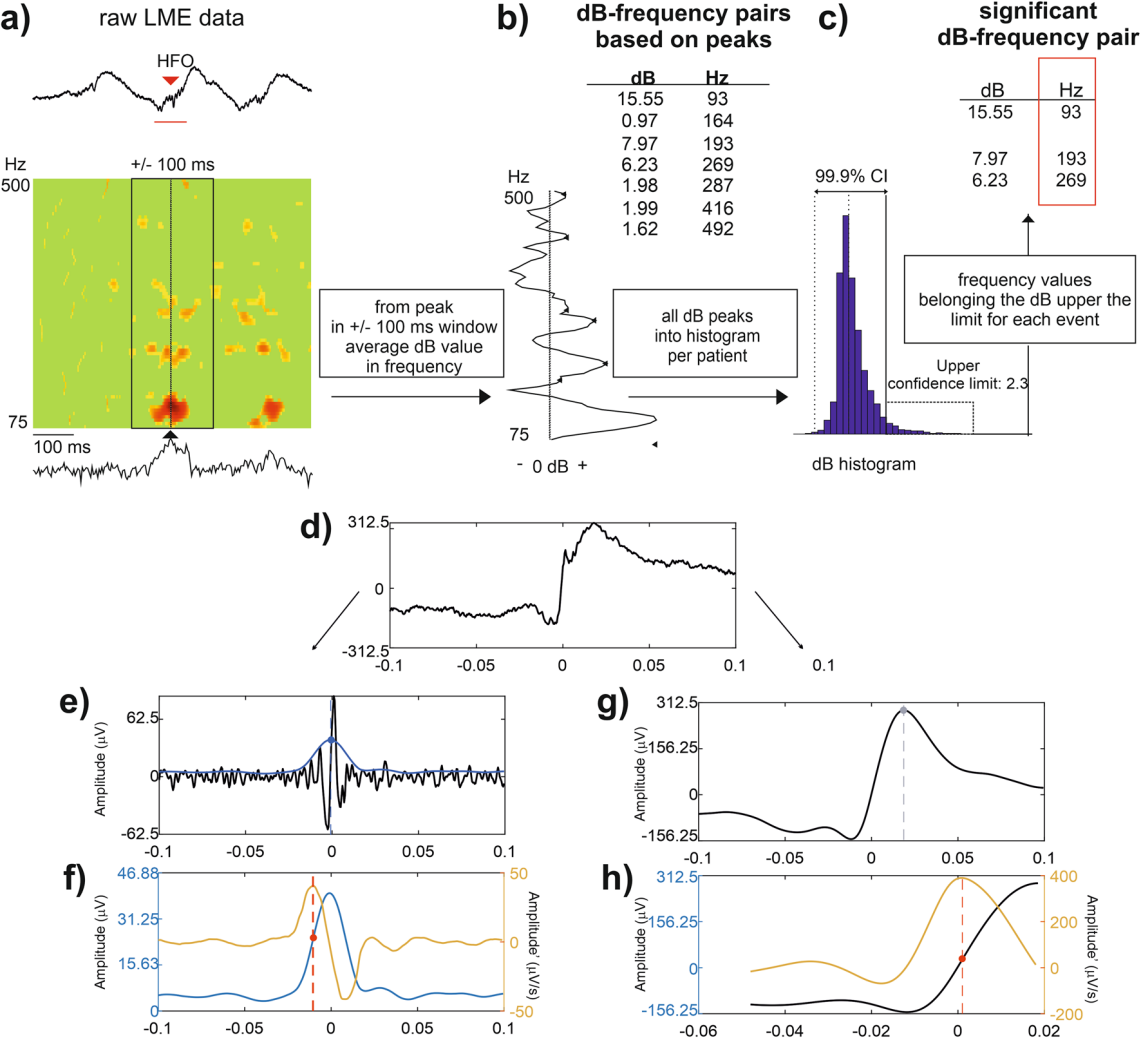
Demonstration of data analysis flow. (**a**–**c**) Analysis of individual HFO frequencies. (**a**) Non-averaged ERSP analysis (bottom colormap) of the raw LFP signal (upper line). ERPS map was frequency averaged (bottom line) to detect highest power time point (marked with black triangle). A 200 ms time window was selected around this time point (black square in the colormap). (**b**) Time averaged ERSP map within the 200 ms window resulted in local power maxima (marked with multiple black left pointing triangle). Frequencies and corresponding deciBel values were collected. (**c**) All events’ all frequency-dB values were collected in one patient on one channel and a dB histogram was built. All dB values below 95% of the histogram were rejected. The remaining frequency-dB values were used for further frequency histograms (see Fig. 3g,h). (**d**–**h**) Calculation of HFO (right column) and IED-like peak (left column) time onset, corresponding to the steepest slope of the events. (**d**) Time windows of ± 100 ms around the peak amplitude of HFO events were selected. (**e**) The raw data was band-pass filtered at 80–500 Hz for the detection of HFO time onset (black curve). First, the RMS of the signal was calculated (blue curve). Blue dashed line marks the peak of RMS data. (**f**) Data was shifted according to RMS peak (± 100 ms around this peak was selected). Red dashed line marks the local maxima (inflection point) of the derivative, representing the steepest slope of the signal (red dot). The time of the steepest slope was considered as the time onset of the event. (**g**) The raw data was band-pass filtered at 2–30 Hz. The gray dashed line marks the peak of this filtered signal. (**h**) Data preceding the peak was used to calculate the derivative of the signal (orange curve). Similarly, as previously, the time onset was found by detecting the local maxima of the derivative, representing the steepest slope of the signal (red dashed line and dot).

1. Continuous time frequency decomposition was used to detect the ongoing variances of high frequency content of the IED associated field potential gradients using Morlet wavelet transformation (80–500 Hz)^58^.
2. Event based frequency analysis for IEDs and HFO peaks (Fig. 1a–c) using only one distribution-based threshold. The high frequency content of the marked events, either IEDs or HFOs, was measured event by event, based on non-averaged event-related spectral perturbation (ERSP)-maps (using pop_newtimef function of EEGLAB)^52^. ERSP parameters were set as: baseline: − 500 to − 200 ms in each event, bootstrap significance: 0.001, analysis frequency 80–500 Hz, wavelet cycle numbers: [3 0.5] ERSP results were given in decibel relative to the baseline activity. Maximum dB high frequency ERSP time point was selected based on wide range (80–500 Hz) frequency average of the ERSP map. All HFOs and IEDs were included in the analysis, separated independently of their co-occurrence (for example the HFO group contains events co-occurring and not co-occurring with IEDs).

Frequency measurement time window was set to − 100 to + 100 ms relative to this maximum. Average frequency content in this window was calculated using time averaged ERPS values of the event. All the local peaks were detected in this average frequency curve. Peak frequencies and corresponding decibel values were stored and collected for all the events. The collected decibel values were analyzed as a distribution and plotted on a dB histogram (with bin size = 40). Individual decibel threshold was set based on the upper 5% of this histogram. All the frequency peaks associated with intensity lower than this threshold were rejected. The remaining frequency peaks were collected as the measures of significant frequency content of the marked events.

This analysis was repeated for all the recording channels, and channel versus frequency histograms were calculated based on the detection occasions falling in 10 Hz bins on each channel for each type of events in each patient.

Gaussian mixture model (GMM) with 2 components (fitgmdist and cluster built-in MatLab functions with default parameters) was applied to find frequency-channel clusters in case of both IEDs and HFOs (Fig. 3i,j). Frequency values were weighted according to the normalized peak frequency distribution maps calculated previously, which were then assigned to matching channel indices. Cutoff values were calculated by averaging frequency values at the edges of the resulting clusters (maximum of first–minimum of second cluster).

### Timing of HFO and IED-like components

To determine the timing of the HFO the steepest slope of the HFO envelope was used (Fig. 1d,g,h)). The HFO envelope was found by calculating the RMS in 5 ms window (11 points) of the band-pass (80–500 Hz) filtered signal. The steepest slope (inflection point) was determined by selecting the local maxima of the first derivative of the HFO envelope in time. If the same event was detected on multiple channels, the onset time of the event recorded in the channel with the highest amplitude was taken into consideration.

Timing of HFOs was compared to the timing of IED-like peaks, detected as the peak of 2–30 Hz band-pass filtered data (in both cases zero-phase shift 4th order Butterworth digital filter was used), ± 100 ms around the HFO peak. IED-like peaks were used instead of originally detected IED events as these also include subthreshold spikes. In this way, we were able to evaluate the timing differences for each channel separately, comparing events arising from the same channel and same time window. The same procedure was applied to compute the time onset of IED-like peaks as previously, except that only the region before peak amplitude was included (Figure d,e,f).

## Results

### Characteristics of the patients

Microelectrode arrays were placed in 44 patients with intractable focal epilepsy; 24 were excluded due to poor data quality (n = 15) (see examples in Supplementary Fig. 1) or due to lack of IEDs and HFOs on the LME (n = 9). We performed further analysis in the remaining 20 patients (17 recordings from neocortex and 3 recordings from hippocampus) where IED detection was successful. We found HFOs in 7/17 patients with neocortical recordings and 2/3 patients with hippocampal recordings (Table 1 summarizes the recordings, Table 2 the patient characteristics).

**Table 1:** Summary of recordings.

	Total number	Good quality	IED	IED + ripple
CTX	41	26	17	7
Hippocampus	3	3	3	2
Summary	44	29	20	9

Numbers indicate the recording numbers in one category as a subset of the recordings in the previous category on the left. There were no recordings with ripples and without IED neither in the CTX nor in the hippocampus.

*IED* interictal epileptiform discharge, *CTX* neocortex.

**Table 2:** List of patients with ripple oscillations detected on LME recordings.

Pt	Lobe	Lat	Etiology	Sex	Age @ OP	55IED rate (/min)	Relationship to focus	Ripple rate (/min)
1	Occipital	L	FCD	F	29	4.09	Near	6.68
2	Frontal	L	Ischemia	F	12	13.85	Near	11.66
3	Parietal	R	FCD	F	41	4.73	Inside	3.71
4	Occipital	R	Unknown	M	43	3.80	Inside	10.38
5	Frontal	R	Oligoastrocytoma	M	42	3.82	Inside	2.12
6	Frontal	R	Unknown	M	24	0.78	Near	0.61
7	Frontal	R	Postencephalitis	M	33	0.78	Inside	6.84
8	Hippocampus		Unknown	F	27	2.14	Inside	3.18
9	Hippocampus	L and R	Dysplasia	M	37	4.78	Inside	1.24
AVG						4.31		5.16

*FCD* Focal cortical dysplasia, relationship to the focus: inside: < 2 cm; near: 2–5 cm; far: > 5 cm distance from the clinically identified seizure onset zone.

In 121 min of recordings in the 9 patients presenting both types of events, 528 HFO and 434 IED events were detected. HFO occurrences averaged 5.16 ± 3.74/min [range 0.61–11.66]; the average rate of IED occurrence was 4.31 ± 3.68/min [range 0.78–13.85; (t-test, p = 0.83).

Recordings were made from all cortical lobes: frontal (n = 3), temporal (n = 3, including neocortical and para-hippocampal), parietal (n = 1) and occipital (n = 2) cortices. Based on subsequent determination of the SOZ by independent clinical evaluation (combined electrophysiological, neuroimaging and clinical data), microelectrodes were either within (within 2 cm, n = 7), or outside (n = 2) of the SOZ.

### The association between HFOs and IEDs is variable

HFOs might co-occur with IEDs but the coincidence varied. Across the nine patients with HFOs and IEDs, an average of 50.1 ± 26.6% [range for patients: 6.1–100%] of the detected HFOs coincided with IEDs within a time lag of 100 ms. The converse relationship (% of IEDs with co-detected HFOs) was 57 ± 27.3% [range 22.2–97.1%]. If we sub-selected the hippocampal recordings (n = 2) a higher proportion [65.4–100%] of HFOs were associated with IEDs.

**Figure 2:**
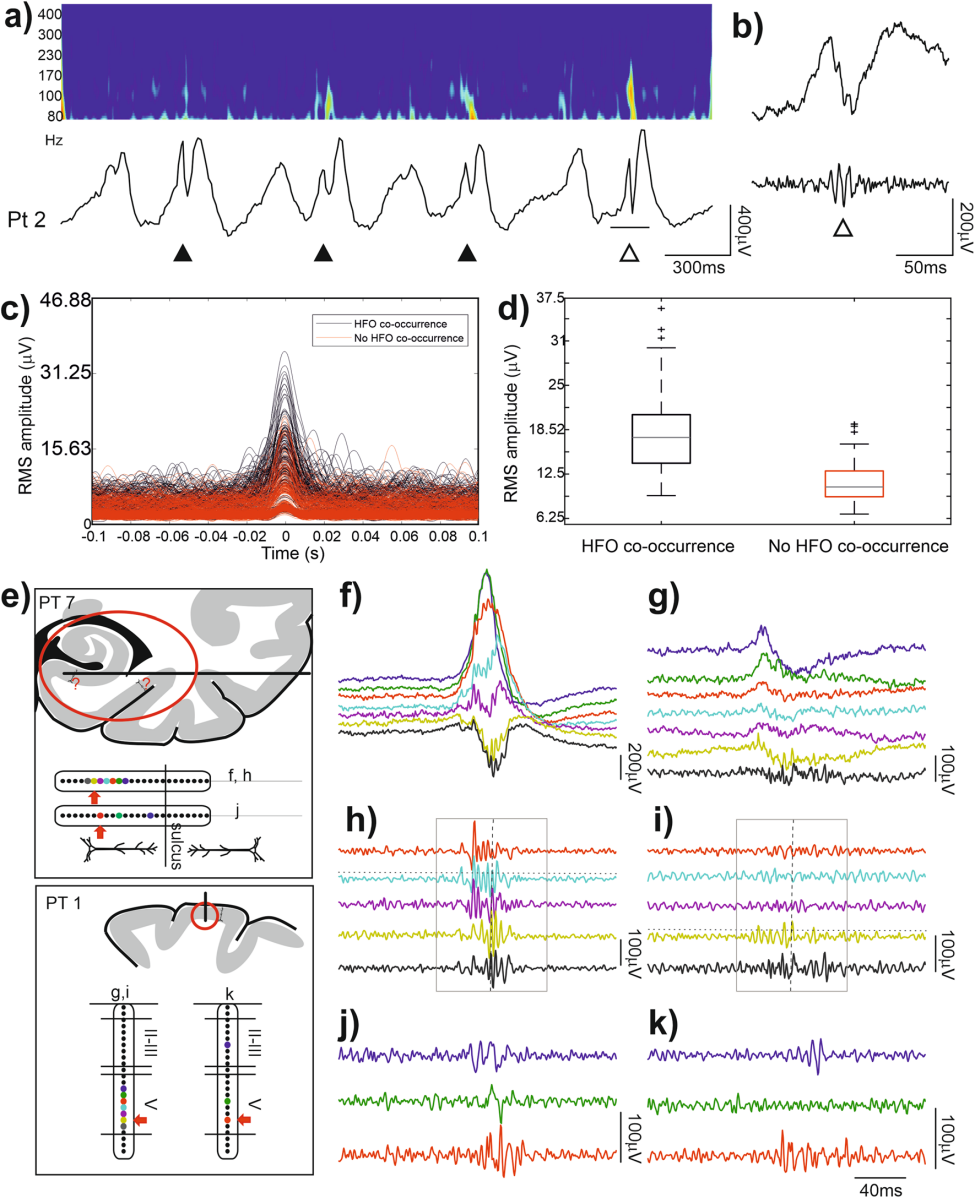
Variability of HFOs in different regions. (**a**) Bottom: Example of ongoing LFP activity. Band-pass filtered (0.5–30 Hz) field potential gradient recording from layer II–III of the neocortex. Black triangle indicate detected IEDs. Top: continuous wavelet transformation of the original signal in the 80–500 Hz range. Note the variability within the 100–130 Hz range among IEDs. The difference is not simply a function of the amplitude of the sharp component of the discharge. Maxima is located at the transition between the IED's positive and negative deflection (positive upwards). Note that only the underlined IED marked with white triangle contained a high frequency event that had been called ripple based on visual detection. (**b**) Top: Unfiltered portion of the underlined LFP trace marked with white triangle in (**a**) Bottom: Band-pass filtered (80–500 Hz) waveform of the same segment. (**c**) Band-pass filtered (80–500 Hz) and root-mean-squared IEDs, aligned to peak RMS amplitude. Peak RMS values of the filtered data were found in the ± 100 ms time window relative to the originally detected IED events. IEDs co-occurring with HFOs are marked with black, while IEDs without co-occurring HFOs are marked with red. (**d**) RMS peak amplitude values of bandpass filtered (80–500 Hz) IEDs. Boxplots represent median with interquartile range (IQR), whiskers mark the most extreme values within 1.5xIQR. Crosses mark outliers outside this range. Color-coding is the same as in (**c**). (**e**) Schematic diagram of recording sites. Top panel: Estimated location of deep laminar multielectrode in the mesial temporal structures. Bottom panel: Thumbtack multielectrode in the occipital lobe. Insets indicate the approximate location of the recording contacts compared to electrophysiologically identified landmarks. (see “Methods” for details). Colored contacts correspond to the colored line on the right-hand side of the figure according to the letter of the actual panel indicated near the inset. Fast ripples were detected on the channel indicated by a red arrow. (see “Methods” for details). (**f**,**g**) Examples of fast ripple associated IED (high-pass filtered at 2 Hz) in the mesial temporal and neocortical recording sites respectively. (**h**,**i**) Same IEDs as in (**f**,**g**) with band-pass filter 80–500 Hz. Note that the neocortical fast ripples with lower amplitude appeared on fewer channels and—especially on the wide band filtration—are more irregular, less sinusoid-like, and appear to contain solitary neuronal events in addition to the field potential oscillation. Horizontal dashed lines above the channel with maximum HFO amplitude mark the 3SD threshold (derived from RMS of band-pass filtered data) in HFO selection criteria (note that the presented data on this figure is not RMS rectified). Vertical dashed lines mark peak amplitude in detected events. Gray boxes represent ± 100 ms windows used for further analysis. (**j**,**k**) Additional examples of IEDs with fast ripple in the depth and slow ripple on the more superficial electrodes in second laminar probes implanted in the mesial temporal and neocortical recording sites respectively. Note that the fast ripple in deep cortical layers precedes the slow ripple in more superficial layers of the neocortex, while the timing is reversed in the mesial temporal lobe.

Certain neurobiological processes underlying visible oscillatory activity in this frequency band may also give rise to non-oscillatory power changes, such as multiple synchronously firing neural groups discharging with variable phase relationship to each other, causing destructive interferences in the recorded extracellular potential^59,60^. Hence, we performed continuous time frequency analysis on the field potential gradient signal of all IEDs to study the variability of the high frequency power (Fig. 2a–d). We found that high frequency content differed from one discharge to another, and that only a subset of discharges coincided with visually detectable HFOs, while the others only showed an increase in power in the 80–500 Hz frequency range. See Fig. 2a,b for demonstrating the phenomenon on a raw signal pattern. The variability of high frequency content of IEDs is also apparent on the representation of band-pass filtered (80–500 Hz) discharges aligned according to the peak of filtered, RMS data (Fig. 2c). Peaks were detected in ± 100 ms window around the originally selected IED events (see “Methods”). Figure 2c also illustrates that there is a considerable subset of IEDs containing “subthreshold” high frequency activity (i.e. not labeled as HFO during the process of HFO detection). Figure 2d highlights the overlap between the peak amplitude distribution of filtered IEDs with or without coinciding HFOs.

### Comparison of HFOs detected in the neocortex and mesial temporal lobe

HFOs were detected visually on the LME data, and wave parameters were calculated automatically. Wideband analysis showed that hippocampal HFOs are significantly shorter in duration (39.3 vs 112 ms), higher in amplitude (8.5 vs 3.5 z-score) and instantaneous frequency (129.1 vs 42.9 Hz) compared to neocortical HFOs (Wilcoxon rank sum test, p < 0.001; Table 3). It has to be noted that the instantaneous frequency detected in the case of neocortical data is below the lower edge of the pass-band filter applied during HFO detection, hence the completed over-threshold cycle number-based method failed to detect the dominant frequency of the oscillation. This finding suggests that neocortical HFOs contained non-uniform, non-sinusoidal cycles with varying amplitudes.

**Table 3:** Wave parameters of ripple oscillations (median [interquartile range]).

	Duration (ms)	p-value	Amplitude (z-score)	p-value	Instantaneous frequency (cycles/duration)	p-value	Ripple density/min
HC—wb (n = 2)	39.3 [28.8–112]	2.8 × 10^–42^	8.52 [6.1–11.9]	2.2 × 10^–129^	129.1 [66.7–169.7]	3 × 10^–74^	15.6
CTX—wb (n = 7)	112 [59–164.5]		3.45 [2.6–4.6]		42.9 [27.1–73.2]		6.8
HC—fast (150–500 Hz)	25 [18.5–37.6]	7.7 × 10^–13^	7.9 [5.5–10.7]	1 × 10^–25^	204.6 [125.5–244]	1.8 × 10^–18^	8.2
CTX—fast (150–500 Hz)	86 [41.9–148.5]		3.43 [2.8–4]		55.1 [34.8–101.3]		0.7
HC—fast (200–500 Hz)	20 [15.9–30]	5.3 × 10^–6^	7.91 [5.6–10.6]	5.6 × 10^–9^	236.8 [173.4–285.7]	6.1 × 10^–7^	6.4
CTX—fast (200–500 Hz)	95.5 [49.5–137.5]		3.3 [2.6–4.1]		54.2 [38.9–90]		0.2
HC—fast (250–500 Hz)	15.5 [13–25.5]	2.2 × 10^–3^	7.6 [5.8–10.2]	1.5 × 10^–5^	277.9 [225.8–324.3]	1.2 × 10^–3^	3.9
CTX—fast (250–500 Hz)	76 [46–135.5]		3.6 [2.9–4.3]		73.4 [35.7–98.6]		0.1

Ripple parameter differences between the hippocampus and the cortex. Statistics were calculated with Wilcoxon rank sum test, except ripple density which was tested statistically.

*HC* hippocampus, *CTX* neocortex, *wb* wideband (80–500 Hz) HFO; fast: fast ripples (sub-selected from the originally identified HFOs, using multiple band-pass filters at [150–500 Hz], [200–500 Hz], and [250–500 Hz]).

Fast ripples were sub-selected from the originally identified events using multiple band-pass filters at [150–500 Hz], [200–500 Hz], and [250–500 Hz]. In the neocortex, fast ripples appeared with smaller amplitude than in the hippocampus (3.4 vs 7.9 z-score at 150–500 Hz). The rate of occurrence of these HFOs decreased differently in the two regions as the lower frequency limit was increased for detection. The hippocampus contained more HFOs in all conditions. The differences of detections/min rates started from 15.6 vs. 6.8 for the 80–500 Hz wideband and ended up in 3.9 vs 0.1 for 250–500 Hz band in the hippocampus and the neocortex respectively. See Table 3 for details. Parallel with this decrease in the number of detected events, the instantaneous frequency was found to be non-concordant relative to the band pass filters in the case of neocortical events (55 Hz @ 150–500 Hz; 54 Hz @ 200–500 Hz, and 73 Hz @250–500 Hz). Unlike this finding, the hippocampal fast ripples resulted in progressively increasing instantaneous frequencies with the increase of the filter’s frequency (see Table 3). This observation also reflects that, compared to hippocampal HFOs, neocortical HFOs contained more asymmetric cycles with varying amplitudes.

### Transmembrane current, multiple unit firing and spectral power distribution of IED and HFO across cortical layers

#### Fast ripple CSD shows an alternating sink-source pair in lower cortical channels than slow ripple CSD

**Figure 3:**
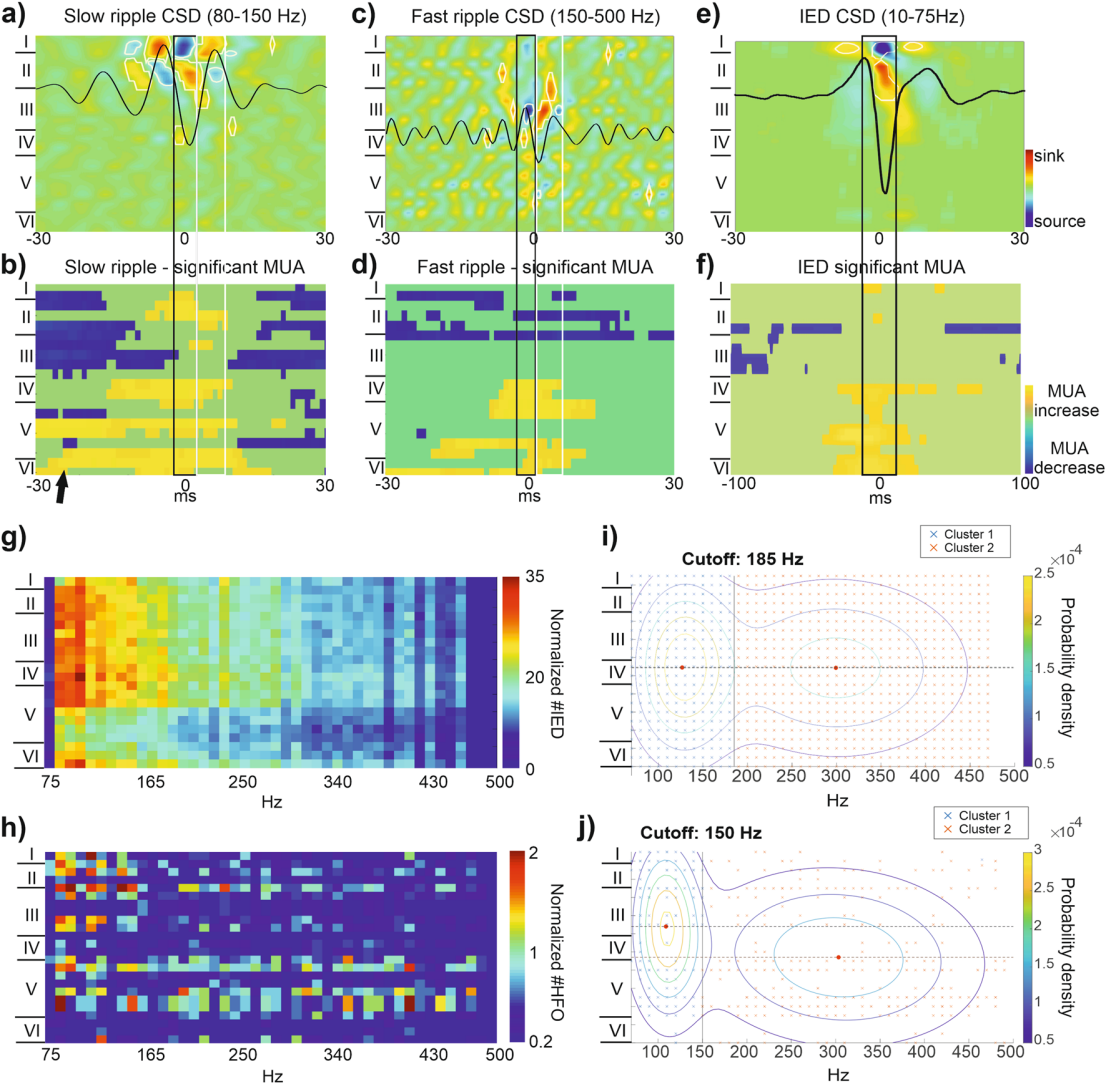
Features of neocortical recording with ripples and IEDs. (**a**–**f**) CSD and MUA analysis of slow ripples, fast ripples and IEDs. In both cases, changes were considered significant if falling outside the ± 5SD range. MUA values within this range were blurred with green, while significant CSD changes were marked with white contour. (**a**) Cross-patient averaged CSD of slow ripples (80–150 Hz), of the 7 patients with detected HFOs on the neocortex The CSD data represents sink (red) and source (blue) amplitudes. Note the phase reversal in the CSD map in the area of the approximated 2nd cortical layer, and the sudden drop of ripple CSD underneath layer II. Black CSD curve shows averaged waveform from channel 5 (750 μm, Layer II–III border). (**b**) Averaged MUA (500–1000 Hz) corresponding to CSD activity of slow ripples. Unlike the CSD, the MUA showed wider activation during the layer II sink (black frame) including the layers I–II and layer IV–VI. Note a significant high frequency activation in the very bottom layers (arrow) preceding the upper layer MUA activation. The activation phase was followed by an inhibition expanding over wide cortical area, however this did not coincide with layer II source (white frame). (**c**) Averaged CSD pattern of the same n = 7 patients for fast ripple components. Note the alternating sink-source pattern is less self-similar, contain only one full cycle in the average signal. The deeper located sink during the highest amplitude part (black box) is located in the granular layer (layer IV). Corresponding CSD trace is indicated with the black curve in the appropriate layer. (**d**) MUA average for fast ripples. Note the similar deep layer activation compared to slow ripples, (**b**) panel, without the supragranular MUA activation. Also, the post activation decrease is missing. (**e**) Averaged CSD map of the IED discharges of the same n = 7 patients. Black CSD curve is selected from the largest amplitude channel 5 (750 μm, Layer II–III border). It is notable that the alternating sink-source-sink pattern (black frame) extended deeper than the HFO, reaching the layer III. (**f**) Averaged MUA for IED according to panel e. Similarly, to fast ripples, average MUA increase was confined to the infragranular layers (below layer IV). (**g**) Frequency distribution histograms plotted as color coded maps measured for all IEDs across cortical layers. This figure includes all patients with detected IEDs on the neocortex (n = 17). Frequency is binned at 10 Hz precision. Each line of the map corresponds to the recording channels. Approximated cortical layers are indicated on the left-hand side. Note the separation of the distribution pattern at channel 15 (2200 µm cortical depth; approximately the area of layer V). Both domains contain mostly low frequency components (< 150 Hz). (**h**) Frequency distribution measured for all neocortical ripple oscillations (n = 7). Note the upper layer 100–150 Hz, and lower layer (beneath channel 10; 1500 µm cortical depth; approximately layer IV) 200–400 Hz peaks. (**i**) Peak frequency clusters, derived from normalized frequency distribution map at (**g**), using gaussian mixture model (GMM). Peak frequencies are marked with crosses, different colors represent different clusters. Probability distribution of clusters is marked by contours. Mean of probability distribution is indicated by red dots. Horizontal dashed lines highlight the cortical layer coinciding with the mean. The vertical line separates the two clusters at the cutoff frequency value. (**j**) Peak frequency clusters, derived from normalized distribution map at (**h**), using GMM. Note that, compared to IEDs, HFOs present lower cutoff frequency (150 Hz). In addition, however the analysis was optimized to the frequency separation, the mean of the slower HFO cluster falls at higher layers (around layer III), while the faster cluster at somewhat lower, indicating the existence of layer separation of the different frequency components.

One strength of the laminar arrangement of microelectrodes is that CSD analysis can be performed showing the location of transmembrane sinks and sources for a defined event. Figure 3a,c shows the grand average CSD pattern of the 7 patients with detected HFOs in the neocortex, in a time window ± 30 ms relative to HFO peak. The CSD maps of slow ripples (80–150 Hz) showed phase reversal around the third intracortical channel (450–500 μm) that corresponded to a sink-source pair in the upper cortical layers. In turn, in the CSD of fast ripples (150–500 Hz), phase reversal was found lower, around channel 11, which corresponds to the limit between infragranular and supragranular layers. Sinks and sources involve mostly the supragranular layer, but also engage the lower cortical domain, extending to layer V.

CSD under IEDs of the same 7 patients similarly showed supragranular sinks and sources but the involved cortical layers extended as deep as layer III (in this case average CSD is visualized in a time window of ± 100 ms on Fig. 3e).

#### Neuronal firing, measured by multiunit analysis during high frequency activity

We recorded multiunit activity (> 500 Hz) simultaneously on the electrodes and averaged according to the peaks of HFOs (Fig. 3b). Layer II sink of slow ripples coincided with layer I–II–IV MUA increase that was slightly preceded by an increase in the MUA activity in the deep layer V (black box and black arrow at Fig. 3b). In the alternating phase, during the layer II source there was still increased MUA in layer II, IV and V, most pronounced in the deep layers (white box at Fig. 3b). Following this, we have found a wide range decrease in the activity of cortical MUA. In the case of fast ripples (Fig. 3d), layer III sink was also associated with increased MUA, appearing first in the bottom layer and lasting also through layer III source. However, here MUA activation was confined to infragranular layers, while supragranular layers presented decreased MUA during the whole examined period.

Compared to slow ripple associated MUA, fast ripple and IID MUA were mostly confined to the infragranular layers (Fig. 3f).

Taken together, these findings reveal that fast ripples localize in deeper cortical layers compared to slow ripples and IEDs. Additionally, the results raise the possibility that the activation of infragranularly located neural populations are related to both HFOs and IEDs, although they do not seem to oscillate in the frequency range of ripples.

#### Spectral distribution within the neocortex reveals supra: and infragranular separation

Based on our previous observation that similar IEDs may harbor varying high frequency content often remaining undetected by visual analysis (see Fig. 2), we performed time–frequency analysis on all the detected HFO and IED events. We therefore analyzed the cortical distribution pattern of the high frequency content in 17 patients (all contained IEDs) and we repeated the analysis for the HFO events of the 7 applicable patients. We omitted the hippocampal recordings from this analysis where the LME position was highly uncertain compared to the lamination.

We used individual event-based analysis of significant frequency peaks to determine the coinciding high frequency content within and across the recording channels (see “Methods” and Fig. 1a–c for details).

The distributions of the frequency peaks (80–500) over the channels are presented in Fig. 3g,h as color coded histograms. We found that the upper cortical layers, above channel 10 (1500 μm) in the HFO group (Fig. 3h), and above channel 15 (2200 μm) in the IED group (Fig. 3g) contained mostly lower frequency (lower than 150–180 Hz) components. In HFO group (Fig. 3h) the frequency peaks of lower layers appear to be more widely and normally distributed compared to upper channels, thus comprising a higher amount of very high frequency content (above 250 Hz). We divided the cortical thickness in the HFO dataset into upper and lower layers, separated at the 10th channel (1500 μm depth) and found that 61% of the slow (lower than 160 Hz) activities were located in the upper layers, while 64% for the fast frequencies were grouped in the lower layers (p < 0.001, Fisher exact test).

Based on peak frequency distribution maps, GMM cluster analysis (Fig. 3i,j) revealed that the high frequency content of both HFOs and IEDs can be divided into two partially overlapping, but clearly separated subgroups. Clusters are defined mainly by frequency, but to a lesser extent also by cortical layer (HFO group). In the HFO group, the first cluster represents frequencies below 150 Hz, where the mean of the cluster’s probability density function falls at 109 Hz (SD 24.2 Hz) and channel 9, corresponding to cortical layer III (SD 6 channels). The second cluster contains frequencies above 150 Hz, where the mean falls at 303.3 Hz (SD 99 Hz) and channel 13, corresponding to cortical layer IV (SD 6 channels). In the meantime, the IED group displays a higher cutoff frequency between the two clusters (185 Hz), and no difference between the channels corresponding to means: channel 11, around layer IV (SD 6 channels). In terms of frequencies, the first cluster’s mean of the IED group falls at 126.5 Hz (SD 35 Hz), while the second cluster’s mean at 299.3 Hz (SD 94 Hz). In both IED and HFO groups, clusters containing faster components present wider distribution in the frequency domain.

We calculated the simultaneous intracortical involvement of significant high frequency peaks and found that they can appear on 5 ± 3 channels (range 1–14) on average for HFO group (n = 7 patients) and on 5.3 ± 4.1 channels (range 1–23) in IED group (n = 17 patients).

#### Time onset differences in deep versus surface events

**Figure 4:**
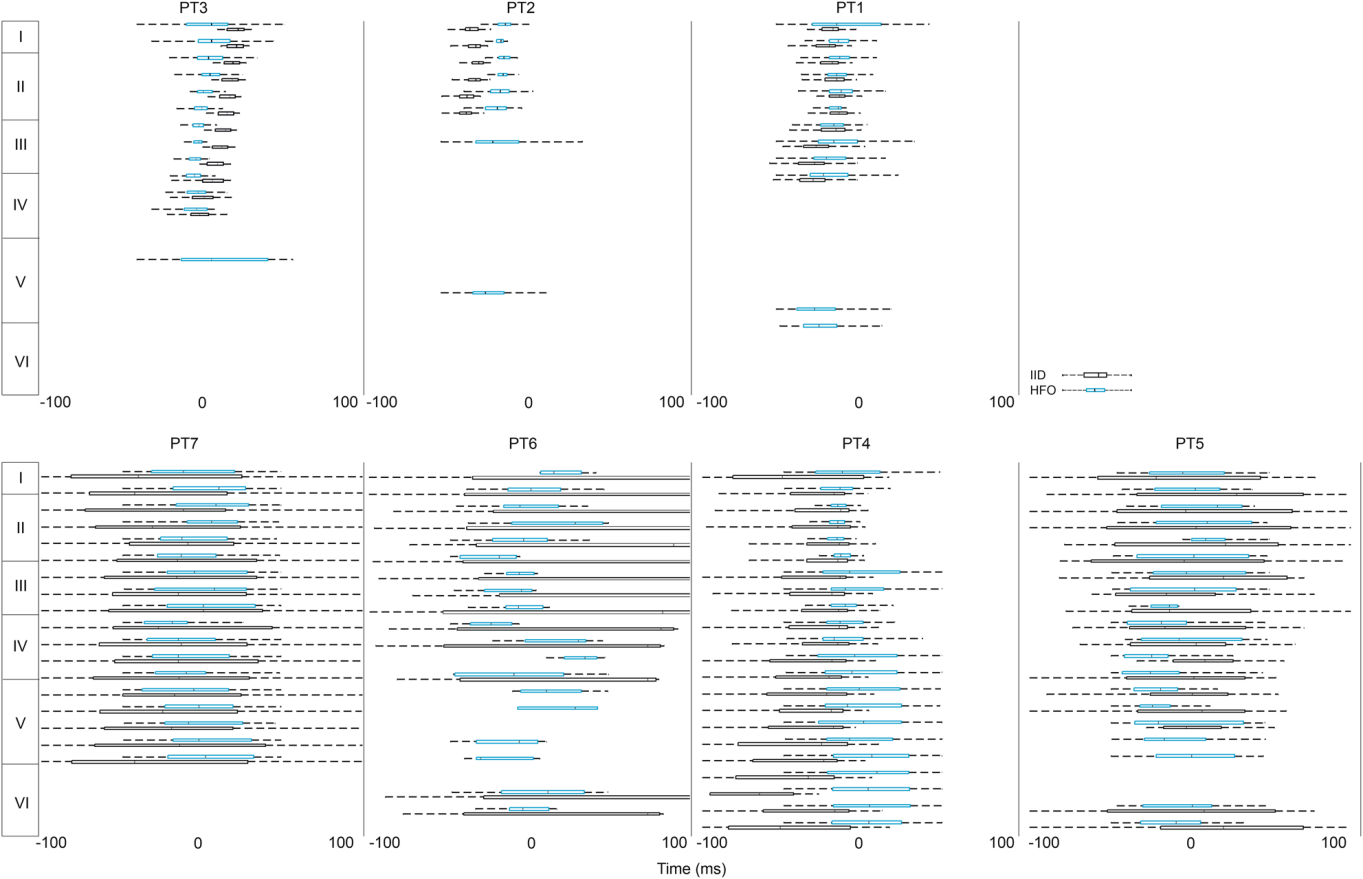
Intracortical timing of IED-like peaks and HFOs. Structured laminar timing of HFO (blue bold outline) and IED (black narrow outline) appearance in 7 subjects. Note the progressive advancement of HFO and IED-like peak timing with deeper and deeper cortical layers in the first 3 patients (top row). Box-whisker plots are plotted as median with interquartile range (IQR), whiskers mark the most extreme values within 1.5xIQR.

First, we have compared the time onset of HFOs across channels, where time onset denotes the time of the steepest slope of the RMS envelope of high frequency spectral filtered HFO data (see “Methods”, Fig. 1d). In 3 out of the 7 patients a reproducible lag was observed, with lower channels preceding the upper channels (blue boxplots on the first row of Fig. 4). This shift was observed both within the upper cortical domain (channel 1–11: 0–1600 µm; layer I–III) and between the domains, as deeper components seemed to precede the upper ones. Nevertheless, in the larger subset of cases (blue boxplots on the second row of Fig. 4), the temporal relationship between upper and lower layers was less consistent.

Then, we have compared the timing of HFOs and IED-like deflections in every channel (similarly, in both cases the timing is represented by the steepest slope time point). The relationship with the timing of these low frequency peaks (black boxplots on Fig. 4) was variable, with HFOs either preceding (PT1) or following (PT2) them. In 4 cases a more random pattern was observed both in terms of laminar relationship and interaction between different events (second row of Fig. 4).

### Non-LME associated HFOs on ECoG recordings

**Figure 5:**
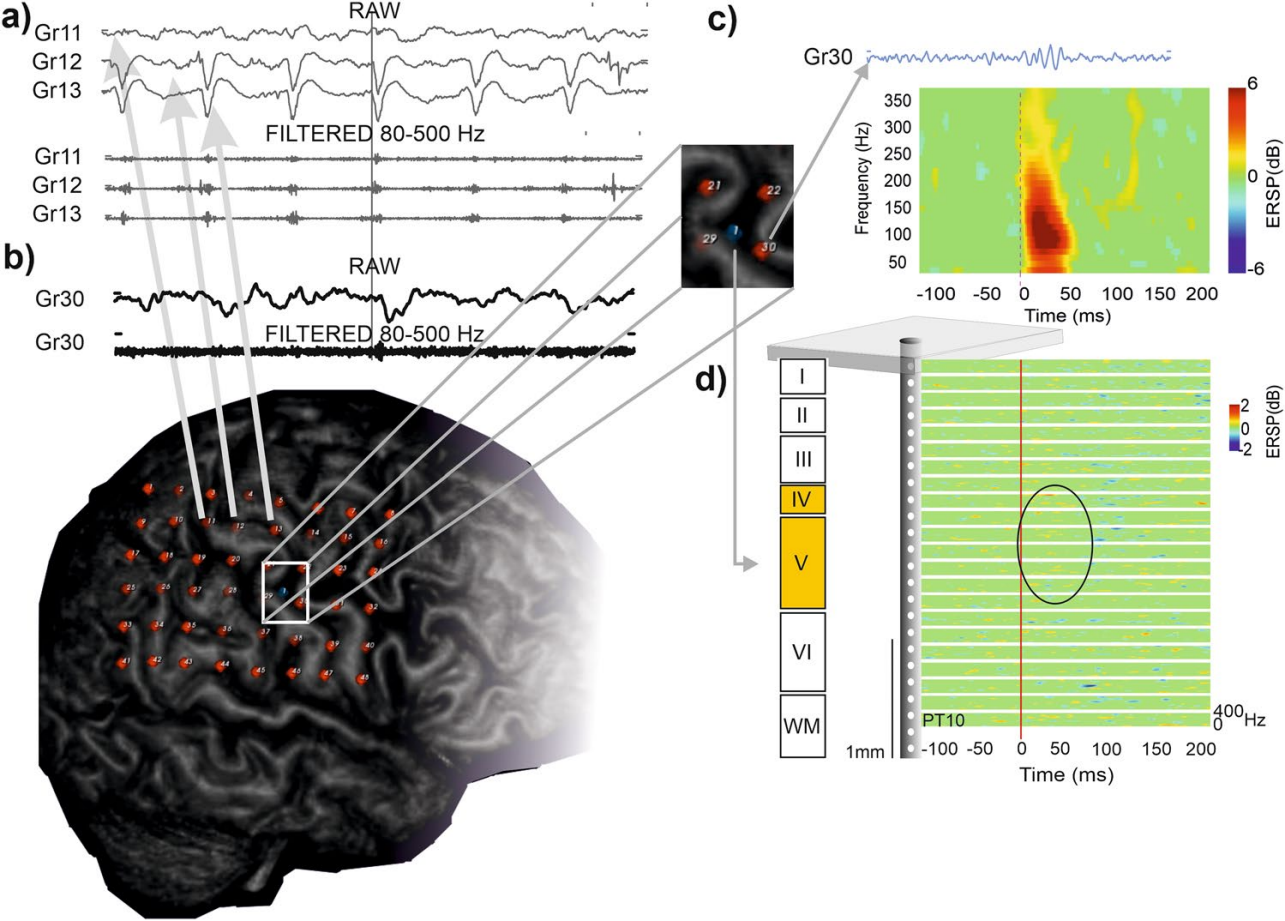
Dissociation of micro- and macrodomain HFO detections outside the seizure onset zone. (**a**) Broad-band and ripple-band filtered EEG (80–500 Hz) from the surface macroelectrodes (grid) from seizure onset zone (Gr11-12–13: see lower panel for electrode locations; macroelectrodes marked with red, LME marked with blue) of 1 patient. This patient had no ripples detected on the LME. (**b**,**c**) Signal of grid 30 electrodes close to LME location showing low intensity high frequency activity (100–150 Hz). Inset shows the location of LME (marked with blue). (**d**) Averaged LME recording during Gr 30 low amplitude ripple oscillation. Surface activity is accompanied by a very slight increase of high frequency activity in layer IV and V (circled with magenta).

To evaluate the relation between the HFOs detected on the LME and on the clinical electrodes, we selected one patient where co-registered grid and LME recordings were available, and no HFOs were detected on the LME. In this case the LME electrode was far (> 3 cm) from the SOZ, yet we found IEDs and HFOs with high amplitude in the SOZ, and IEDs and HFOs with lower amplitude closer to the LME electrode on the surface (Fig. 5). During the identifiable surface HFOs on the grids, no visible HFOs were detected intracortically, even though a small change in the power of high frequency components was observed on the time–frequency analysis of the LME signal in the middle layers. Hence, in this case, surface HFOs appeared both within and outside the SOZ area, while the LME, located outside the SOZ, showed no HFOs. Although we lack microelectrode recordings from the SOZ (due to the technical limitations of microelectrode positioning), these observations might hint at a possible role of LME in SOZ localization, worth considering in further research.

## Discussion

We used laminar microelectrode arrays to explore the intracortical organization of HFOs and IEDs in human focal epilepsy. Our results show that slow and fast ripple oscillations have distinct spatial organizations. In the neocortex, mostly slow ripple oscillations were observed, which took place in the supragranular layers via active local synaptic events, accompanied by alternating, but asynchronous high and low neuronal firing periods throughout the cortex. Fast ripples appeared in lower cortical layers compared to slow ripples and firing neural populations were mostly confined to the infragranular layers demonstrating that different frequency activations can be located in different cortical domains. Hippocampal fast ripple oscillations were higher in amplitude and frequency demonstrating potentially different generation mechanisms.

IEDs occurred with and without HFOs and presented varying high frequency content Roughly half of the activities coincided with each other indicating that concurrence occurs^4^ with variance in the microdomain level.

### Intra-neocortical domains of the ripple and IED generation; separation of different frequencies in space

Our laminar multielectrodes provide insight into the parallel processes across different cortical layers^48^. We subdivided the neocortical depth into 2 domains separated at ~ 1.5 mm depth. According to the general laminar organization of the human cortex, this separation is located around or beneath layer IV (internal granular layer) even in posterior cortices^61^.

The largest currents originated in the supragranular layers both for HFOs and IEDs. The associated MUAs showed more widespread localization for both activities involving mostly the infragranular layers. This finding was concordant with the similar analysis of slow waves in sleep^49^ and the majority of spindle oscillations^45,46^ during non-REM sleep, and with the biggest amplitude components of IEDs^44^.

Nearly all neocortical HFOs were slow ripples (slow vs fast ripple rate: 6.75 vs 0.69/min). Analyzing the distribution of coinciding frequencies across cortical layers, we found that the supragranular layers encompassed mostly slow ripple components, while the infragranular layers showed wider ripple frequency distribution resulting in fast ripple components appearing mostly infragranularly. Cluster analysis strenchtened also that fast components were biased in deeper layers than the slow components. This observation is further supported by the CSD pattern of slow and fast ripples. While slow ripples engaged only supragranular layers (expanding to layer III), fast ripples comprised both cortical domains. Additionally, the highest amplitude sinks and sources of fast ripples were also found in lower layers (at the border between supra- and infragranular layers).

Our previous findings showed that locally generated IEDs had an early infragranular layers sink, while propagated IEDs initiated in the supragranular layers, indicating that the IED genesis is linked to the infragranular layers^43^. Our data are consistent with this observation, since IED associated MUA was rather localized into the infragranular layers (Fig. 3f). Additionally, infragranular MUA precedes supragranular MUA also in the case of detected HFOs (Fig. 3b,d). Regarding that all of our HFO containing samples were recorded within or near the SOZ, this might hint on the pathological nature of these oscillations. However, further analysis is required to prove this theory. Based on recent experiments studying rodent neocortex, sparse neuroglia-form neurons can be found in deep cortical layers that has anticorrelated spiking activity with the vast majority of excitatory and inhibitory neurons and participate in oscillatory phase transitions during slow-wave sleep, facilitating the initiation of the excitatory up-state^62^. The role of these neurons in IED generation is unknown but possible based on their intracortical location and function.

We also counted the number of involved channels in each event, and we found that on average 5 electrodes out of 23 within a given laminar probe were activated together. In our bipolar recordings the 40 µm diameter contacts were separated by 150 µm, so their fields have minimal overlap. We suggest that HFOs characteristically engage multiple levels of the cortex and that the different microdomains may be activated not only side-by side in the cortex^63^ but separated horizontally by layers.

Our results underline the importance of the separate function of cortical layers and domains in epileptogenesis and draw attention to the function of deep cortical layers and infragranular neurons during HFO and IED generation in focal epilepsy.

### Hippocampus versus neocortex

Hippocampal microdomain HFOs appeared different from those in the neocortex. Hippocampal oscillations were shorter, but higher in frequency and amplitude; with robust, high amplitude fast ripples not presented elsewhere. We saw markedly different fast ripple numbers in hippocampus vs neocortex (8.6 vs 0.69/min) and a failure of detecting instantaneous frequency in the neocortex using completed-cycle number-based method (see Table 3). The varying cycle amplitude resulted in a lower number of overthreshold cycles in the RMS window using Staba’s^22^ method. The possible explanation of this result might be that neocortical fast ripples contained non-uniform, non-sinusoid cycles including non-rhythmic unitary events. See Fig. 2 for typical fast ripples in the neocortex and the hippocampus. This finding might point to the nature of detected neocortical HFOs, since it has been hypothesized that pathological HFOs derive either from completely asynchronous firing neurons^64^ or clusters of abnormally synchronized neurons firing at lower frequencies, but with a phase delay between them^21,65^. In animal studies, the neocortex was shown to contain some fast-spiking interneurons, capable of intrinsically discharging at high frequencies (~ 500 Hz), which would most probably generate more sinusoid-like oscillations (Jones 2000). Nevertheless, the existence of these neurons in human brain and their potential contribution to the generation of physiological and pathological HFOs respectively, has not been proved yet.

Rodent^13^ and human^19^ studies reported extensive ripple activity in the hippocampus and relatively low in the cortex. The comparison of hippocampal versus neocortical HFOs showed that the hippocampus contains more uniform, sinusoid-like oscillations with higher amplitudes than the neocortex. These observations suggest that hippocampal fast ripples are generated differently involving more neural synchronization.

### Frequency threshold between slow and fast ripples

The exact boundary for the separation of HFOs into slow and fast ripples is still ambiguous. On one hand, the term “ripple” is used for both slow and wide band oscillations including fast frequencies. On the other hand, the cutoff threshold used to separate fast ripples from slower oscillations varies between publications. In the early reports from Staba and colleagues separated slow and fast ripples between 150 Hz^18,24^ that was a result of histogram analysis of hippocampal activities. Later the authors used increasing limits varying between 200 or 250 Hz^25,39^.

The confusion is even more extended because cortical gamma activity (CG) partially overlaps in frequency. In a subset of the literature 90–150 Hz is called fast gamma^66,67^, indicating that this frequency range is part of the CG spectrum, but different from its average range. CG usually measured in the 30–80 Hz range is related to cognitive processes^68,69^, and represents binding phenomena^70,71^.

As we faced this terminological confusion, we decided to use a wide 80–500 Hz as HFO range that inherently prevented us from differentiating the various types of high frequency oscillations within this spectrum. However, taking into account that CG is longer lasting irregular oscillation that are associated with cognitive processes, we detected time restrained oscillatory packages that are, therefore, different from the average presentation of CG. We have to add that the detected 80–150 Hz oscillation coincided with IEDs that are clearly pathological in contrast with the physiological nature of CG.

The observation that supragranular slow ripple oscillations fell consequently below 150 Hz (Fig. 3h) is supported by cluster analysis, which demonstrated a clear separation between the two resulting frequency-channel clusters at 150 Hz in case of ripples (Fig. 3j). Intriguingly, high frequency content of IEDs proved to be separated at a higher value, at 185 Hz (Fig. 3i).

Hence, we suggest that activities between 150 and 200 can be grouped with fast ripples more than slow ripples. This result also raises the question on the equality of the two approaches, namely analyzing HFOs or IEDs, and indicates that IED associated high frequency content represents partly different activities than the visually detected HFOs.

### Local neuronal network mechanisms of high frequency activity generation

There are multiple investigations examining mechanisms underlying HFOs^6,21,40^. The data presented here suggest that slow HFOs resembled membrane oscillations and were generated locally mostly in the supragranular layers, while the infragranular faster oscillations were often more irregular, producing less sinusoid-like oscillations.

In all cases infragranular population activity preceded upper cortical layers. According to the intracortical information flow, this direction is typically feed-back type^72^. In rats and cats the excitatory feedback from layer V to layer III was extremely low, indicating that a reverberating excitatory loop is unlikely to exist in normal cortex^73^. There is, however, an extensive excitatory and inhibitory neuronal network synchronizing the activity of the layer II/III and layer V mostly studied in animals^74^. Based on data from human slice preparations both bursting pyramidal and synchronized interneurons are involved in generation of epileptiform activities in the cortex^75,76^. It is possible that the initiating action of the ripple generation takes place through these intracortical connections. The interneuronal hypothesis is strengthened by a careful analysis of single unit activities in the hippocampus, showing that the presumptive inhibitory neurons initiate and maintain the synchrony during the ripple oscillation^40^. The supragranular slow ripple oscillation itself seems to harbor both excitatory and inhibitory phases where cortical neurons do increase their firing rate, both supra- and infragranularly followed by a wide cortical inhibitory phase.

Another theory concerning pathological fast ripple generation mentions the modified bursting propensity of neurons in disease conditions, such as elevated extracellular K + concentration in epileptic tissue. In this environment, neurons with similar bursting properties start to fire in synchronous bursts, resulting in ripples emerging on field potential recordings^59,77^.

Our observation that the infragranular activity showed less self-similarity, and no (Fig. 3a) or less prominent (Fig. 3c) average CSD waveform, points to the possibility that some interference patterns take place in that region. Additionally, the apparent lack of MUA synchrony with CSD patterns might also support the explanation of Foffani and collegaues’ concerning fast ripple activity, that the measured high frequency is the sum of activity of asynchronously activated lower frequency generators^21,64,65^.

### Relationship with surface macroelectrode signals and seizure onset zone

We showed that it is possible not to see any ripples on the LME, while successive ripples are present in the overlying surface macroelectrode. We suggest that surface ripples may reflect the summed activity of multiple cortical microdomains, while only some are locally produced HFOs.

Analyzing the interaction of this phenomenon with the LME location relative to the SOZ in one patient, we found that in this case the LME was located farther from the SOZ, suggesting that the intracortically detectable HFOs might be a more specific sign of being within the SOZ, and microdomain registration may be more sensitive for locally generated pathological activities. This conclusion was strengthened by the observation that in all seven patients with intracortical HFOs the LME electrode was placed within the SOZ (Table 2) which can also explain the relatively low number of intracortical ripple detections (7 out of 26 patients).

### Limitations

Our study has some noteworthy limitations. First, due to clinical considerations, histological reconstruction of the electrode penetration in the brain tissue was not always available. Therefore, the sampled cortical depth validation relied on the electrophysiological characteristics of the data derived from previous findings^48^. Additionally, due to the surface tension, the top of the LME, made by a silastic sheet, adheres to the cortical surface. Hence, the depth of contacts below the pial surface is known. Second, due to several ethical and technical restraints, LME recordings can be obtained only in relatively low sample size and there are also limitations in the anatomical positioning of the array. Thus, the evaluation of the role of LME in the SOZ localization is difficult. However, we believe the presented data might serve as a useful reference for future, more extensive studies.

## Conclusion

Neocortical ripple oscillations showed marked difference from hippocampal HFOs, with less fast component. The typical intra-neocortical pattern was characterized by top layer alternating sink-source pattern accompanied by alternating wide cortical neuronal firing increase. The frequency distribution showed intracortical preference with fast ripple components mostly present in the infragranular layers, while the supragranular layers produced mostly slow ripple components. Our analysis proved that cortical domains participating in epileptiform activities are not only separated vertically but also horizontally and underlines the importance of the infragranular layers generating early neural firing activity, preceding ripple oscillation on the superficial layers.

## Supplementary Information

Supplementary Information.

## Data Availability

The data that support the findings of this study are available on request from the corresponding author. The data are not publicly available due to privacy or ethical restrictions. The software used for data acquisition and analysis is freely available on request from the corresponding author.

## Abbreviations

IED: Interictal epileptiform discharge
MUA: Multiple unit activity
DC: Direct current
SD: Standard deviation
HFO: High frequency oscillation
LME: Laminar multielectrode
RMS: Root mean square
CSF: Cerebrospinal fluid
SOZ: Seizure onset zone
IQR: Interquartile range
GMM: Gaussian mixture model
