# Supplementary material for: The role of superficial and deep layers in the generation of high frequency oscillations and interictal epileptiform discharges in the human cortex
Source: Sci Rep. 2023 Jun 14;13:9620. doi: 10.1038/s41598-022-22497-2 (PMC10267175; doi:10.1038/s41598-022-22497-2)

**Supplementary material**

*Supp. Figure 1: Exclusion of patients due to poor data quality*Figures represent the bandpass filtered (80-500 Hz) data of 3 patients presenting high amount of high frequency noise (a, b) and abrupted recording due to technical concerns (c).

High-amplitude spikes on the last channel represent trigger signals sent by the recording software for the co-registration of macro- and microelectrode recordings.


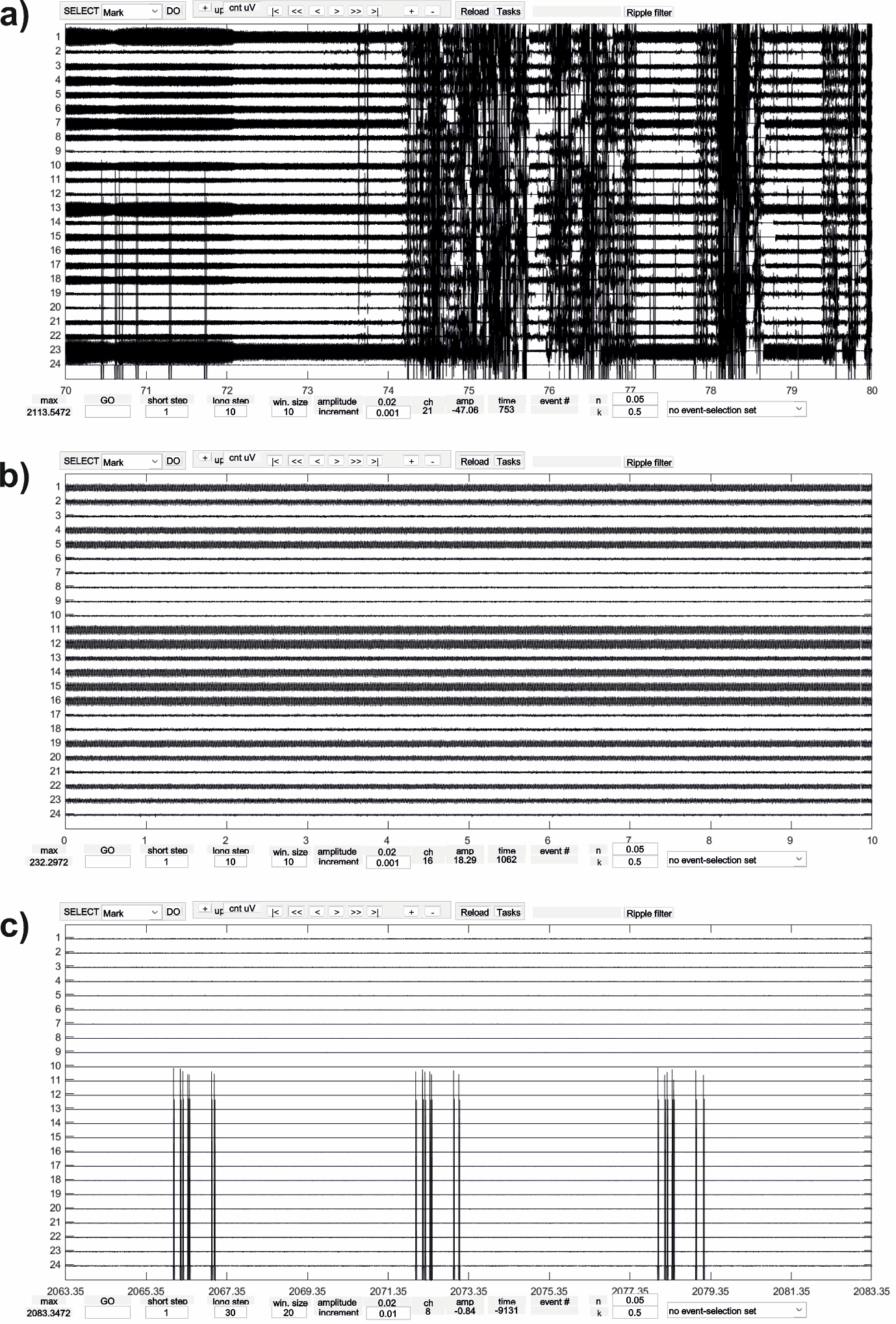

Supplement: Supplementary file 1 — Supplementary Information. [file 41598_2022_22497_MOESM1_ESM.docx]
